# Alirocumab, a Therapeutic Human Antibody to PCSK9, Does Not Affect CD81 Levels or Hepatitis C Virus Entry and Replication into Hepatocytes

**DOI:** 10.1371/journal.pone.0154498

**Published:** 2016-04-26

**Authors:** Aarti Ramanathan, Viktoria Gusarova, Neil Stahl, Anne Gurnett-Bander, Christos A. Kyratsous

**Affiliations:** Regeneron Pharmaceuticals, Inc., Tarrytown, NY, United States of America; UMR Inserm U1052 / CNRS 5286, FRANCE

## Abstract

**Background:**

Proprotein convertase subtilisin/kexin type 9 (PSCK9) is secreted mainly from the liver and binds to the low-density lipoprotein receptor (LDLR), reducing LDLR availability and thus resulting in an increase in LDL-cholesterol. While the LDLR has been implicated in the cell entry process of the hepatitis C virus (HCV), overexpression of an artificial non-secreted, cell membrane-bound form of PCSK9 has also been shown to reduce surface expression of CD81, a major component of the HCV entry complex, leading to concerns that pharmacological inhibition of PCSK9 may increase susceptibility to HCV infection by increasing either CD81 or LDLR availability. Here, we evaluated effects of PCSK9 and PCSK9 blockade on CD81 levels and HCV entry with a physiologically relevant model using native secreted PCSK9 and a monoclonal antibody to PCSK9, alirocumab.

**Methods and Results:**

Flow cytometry and Western blotting of human hepatocyte Huh-7 cells showed that, although LDLR levels were reduced when cells were exposed to increasing PCSK9 concentrations, there was no correlation between total or surface CD81 levels and the presence and amount of soluble PCSK9. Moreover, inhibiting PCSK9 with the monoclonal antibody alirocumab did not affect expression levels of CD81. In an *in vitro* model of HCV entry, addition of soluble PCSK9 or treatment with alirocumab had no effect on the ability of either lentiviral particles bearing the HCV glycoproteins or JFH-1 based cell culture virus to enter hepatocytes. Consistent with these *in vitro* findings, no differences were observed in hepatic CD81 levels using *in vivo* mouse models, including *Pcsk9*^-/-^ mice compared with wild-type controls and hyperlipidemic mice homozygous for human *Pcsk9* and heterozygous for *Ldlr* deletion, treated with either alirocumab or isotype control antibody.

**Conclusion:**

These results suggest that inhibition of PCSK9 with alirocumab has no effect on CD81 and does not result in increased susceptibility to HCV entry.

## Introduction

Entry of the hepatitis C virus (HCV) into hepatocytes (reviewed in Ploss & Evans 2012[1]) requires the interaction of the virus particle with numerous host cell proteins, including the tetraspanin CD81 [2], the scavenger receptor class B type I [3], the two tight junction proteins claudin-1 [4] and occludin [5], glycosaminoglycans [6], and the low-density lipoprotein receptor (LDLR) [7].

Proprotein convertase subtilisin/kexin type 9 (PSCK9) is a protease synthesised primarily in the liver [8, 9] PCSK9 binds to LDLRs, resulting in their degradation, so that fewer LDLRs are available on liver cells to remove excess LDL-cholesterol (LDL-C) from the plasma [10, 11]. Thus, PCSK9 inhibition is an attractive target for treating hypercholesterolemia.

Alirocumab is a fully human PCSK9 inhibitor antibody approved by the FDA as adjunct to diet and maximally tolerated statin therapy for the treatment of adults with heterozygous familial hypercholesterolemia or clinical atherosclerotic cardiovascular (CV) disease, who require additional lowering of LDL-C. In Phase 3 clinical trials, alirocumab at a dose of 75 or 150 mg every 2 weeks reduced LDL-C by 44.1 to 61.0% [12–17].

Over-expression of an artificially engineered, non-secreted, cell membrane-bound form of PCSK9 and the cytoplasmic form of PCSK9 have been shown to modulate expression of CD81, a major component of the HCV entry complex [18, 19]. This raises the concern that monoclonal antibodies that inhibit PCSK9 binding to the LDLR might result in an increase in CD81 levels and an associated augmentation of HCV entry into hepatocytes, thereby enhancing susceptibility to HCV infection [20]. However, the models used to date (which utilize ectopically over expressed, membrane-associated PCSK9 protein) are not physiologically relevant, because native PCSK9 is secreted and not membrane bound. Furthermore, these methods are not suitable for assessing effects of monoclonal antibodies which have no impact on production of intracellular PCSK9 [21]. Thus, a more appropriate model for studying the effects of a monoclonal antibody to PCSK9 on HCV entry is required.

The current study used the native secreted form of the PCSK9 protein in both *in vitro* and *in vivo* models to investigate whether PCSK9 expression impacts CD81 cell surface levels. Objectives were to determine the biological relationship between PCSK9 and CD81, by investigating the effects of the secreted form of PCSK9 on CD81 levels, effects of antibody-mediated inhibition of the PCSK9/LDLR interaction on CD81 levels *in vivo* and *in vitro*, and effects of soluble PCSK9 and antibody-mediated inhibition of the PCSK9/LDLR interaction on HCV entry.

## Materials and Methods

### Proteins

The monoclonal antibody to human PCSK9 (hPCSK9), alirocumab, and an isotype control antibody with irrelevant specification, were developed by Regeneron Pharmaceuticals, Inc. (Tarrytown, NY, USA) using VelocImmune^®^ technology.

Human PCSK9 protein (hPCSK9-MycMycHis) and a human PCSK9 gain-of-function mutant (hPCSK9-D374Y-MycMycHis) were used for *in vitro* assays. Proteins were purified by immobilized metal affinity chromatography (IMAC) followed by anion exchange and size exclusion chromatography.

Anti-mouse CD81 antibody (EAT-2, sc-18877, monoclonal Armenian hamster; Santa Cruz Biotechnology Inc., Dallas, TX, USA), anti-human CD81 antibody (sc-9158, polyclonal rabbit; Santa Cruz Biotechnology Inc.), anti-mouse LDLR antibody (AF2255, polyclonal goat; R&D Systems, NE Minneapolis, MN, USA), anti-human LDLR antibody (AF2148, polyclonal goat; R&D Systems), anti-human transferrin receptor (TfR) antibody (loading controls) that cross reacts with mouse TfR (AF2474, polyclonal goat; R&D Systems), anti-human glyceraldehyde 3-phosphate dehydrogenase (GAPDH) (loading control) that cross reacts with mouse (2118S, monoclonal rabbit; Cell Signaling Technology, Danvers, MA, USA), and anti-mouse actin (loading control) that cross reacts with human (ab3280, monoclonal mouse; Abcam, Cambridge, MA, USA) were used in Western blot analyses.

Anti-hCD81, fluorescein isothiocyanate-conjugated (561956, mouse monoclonal; BD Biosciences, San Jose, CA, USA), anti-human LDLR phycoerythrin-conjugated (FAB2148P, mouse monoclonal, R&D Systems) and the corresponding isotype controls (551436 and 551954, respectively; BD Biosciences) were used for flow cytometry.

The anti-CD81 antibody (clone JS-81) used as a positive control for inhibiting Jc1 HCV infection was purchased from BD Pharmingen, (San Diego, CA)

### Nucleic acids

DNA sequences encoding for HCV genotype 1a (H77) E1E2 glycoproteins corresponding to aa191-746 of the virus polyprotein were synthesized by GeneArt^®^ and cloned into pcDNA3.1 (Life Technologies, Grand Island, NY, USA) to generate pCMV-H77-E1E2. Plasmids psPAX2 and pWPXLd-GFP encoding for HIV gag pol and green fluorescent protein (GFP) in the context of an HIV genome were obtained from the Trono lab (Ecole Polytechnique Fédérale de Lausanne, Lausanne, Switzerland). pCK-retro-luc was constructed by replacing the GFP open reading frame in pWPXLd-GFP with the gene encoding for firefly luciferase. A plasmid encoding encoding the Jc1 genotype 2a chimeric HCV genome [22] in a bicistronic configuration where the HCV IRES directs the translation of the Gaussia Luciferase (GLuc) protein and the EMCV IRES directs expression of a chimeric HCV polyprotein encoding the core-first 1/3^rd^ of NS2 and the JFH-1 remaining NS2-NS5B coding sequence was kindly provided by Charles M. Rice, Rockefeller University (reviewed in Vieyres & Pietschmann, 2013 [23]).

### Tissue culture

Huh-7 cells (human hepatocyte-derived cellular carcinoma cell line; [24]) and 293T cells (human kidney cell lines; American Type Culture Collection [ATCC], Manassas, VA, USA) were grown in high-glucose Dulbecco's modified eagle medium containing 10% v/v fetal bovine serum, supplemented with antibiotics (penicillin/streptomycin) at 37°C in a 5% CO_2_ atmosphere.,

Transfections were performed using Lipofectamine LTX (Life Technologies) following the manufacturer’s instructions.

### HCV pseudoparticles and cell culture virus

HCV pseudoparticles (HCVpp) were generated by co-transfecting 293T cells with a mix of pCMV-H77-E1E2, psPAX2 and pCK-retro-luc. Supernatants were harvested 48–72 hours post transfection, clarified using centrifugation, aliquoted and frozen at -80°C. Huh-7 cells cultured in 6-well plates were incubated with recombinant PCSK9 proteins (wild-type PCSK9 or D374Y mutant) and/or alirocumab or isotype control monoclonal antibody, as indicated, for 6 hours at 37°C. Cells were then harvested using 1% ethylenediaminetetraacetic acid (EDTA) and washed with PBS. 15,000 cells were infected in suspension with artificial HCVpp expressing firefly luciferase and then transferred to wells of a 96-well plate. Infected cells were incubated for 48–72 hours. Infection efficiency was quantitated by luciferase detection with the BrightGlo^®^ luciferase assay (Promega, San Luis Obispo, CA, USA) and read in a Victor^®^ X3 plate reader (Perkin Elmer, Waltham, MA, USA) for light production.

Cell culture derived infectious Jc1 HCV was generated by transfection of Huh-7 cells with in vitro transcribed viral RNA and titres of virus collected in these supernatants were determined by NS5A staining limited dilution assay on Huh-7.5 cells, as previously described [25]. HCV-GLuc stocks were used at 5.0x10^4^-1.0x10^5^ tissue culture infectious dose/mL (TCID50/mL), The HCV polymerase inhibitor 2’C-methyl-adenosine (2’CMA) [26] was provided by Timothy Tellinghuisen (Scripps Research Institute).

### Western blot analyses

Total protein levels of LDLR, CD81, TfR, GAPDH and actin were assessed by sodium dodecyl sulfate polyacrylamide gel electrophoresis (SDS PAGE) and Western blotting analysis. Equal amounts of liver protein extracts from treated animals (50 μg protein) or lysates in LDS Sample Buffer (Life Technologies) from equal numbers of Huh-7 cells were resolved by SDS-PAGE in precast gels (NuPAGE^®^; Life Technologies), transferred to nitrocellulose membranes and probed with the indicated antibodies. Bound primary antibodies were detected with horseradish peroxidase-coupled secondary antibodies against respective immunoglobulin G using an enhanced chemiluminescent detection system (SuperSignal; Thermo Scientific, Lafayette, CO, USA).

### Flow cytometry

Stained cells were washed with Staining Buffer, fixed with 2% paraformaldehyde for 30 minutes at room temperature and analyzed on a Guava^®^ easyCyte flow cytometer (Millipore, Billerica, MA, USA) using the FlowJo^®^ software (Tree Star, Inc., Ashland, OR, USA).

### Assessment of surface and total CD81 and LDLR levels in Huh-7 cells

Huh-7 cells were seeded to approximately 75% confluency on 10 cm plates.

For assessment of surface protein levels, Huh-7 cells were incubated with increasing concentrations of recombinant PCSK9 proteins (5–500 nM wild-type PCSK9 or 0.2–20 nM D374Y mutant) and 300 nM alirocumab or isotype control monoclonal antibody, as indicated, for 6 hours at 37°C. The cells were then washed with phosphate-buffered saline (PBS), harvested using 1% EDTA, extensively washed with PBS and stained with the indicated antibodies in Staining Buffer (BD Biosciences, cat. # 554657) before analysis using flow cytometry.

For assessment of total protein levels, Huh-7 cells were incubated with increasing concentrations of recombinant PCSK9 proteins (wild-type PCSK9 or D374Y mutant, as above) and 200 nM alirocumab or isotype control monoclonal antibody, as indicated, for 6 hours at 37°C. Cells were then washed with PBS, harvested with 1% EDTA, and analysed by Western blot.

### *In vivo* experiments

All animal procedures were conducted in compliance with protocols approved by the Regeneron Pharmaceuticals Institutional Animal Care and Use Committee.

#### Effect of Pcsk9 deletion on LDL-C, CD81 and LDLR

*Pcsk9*^*-/-*^ mice [27] and their wild-type littermates (all males) on chow diet were sacrificed at 9 weeks of age following a 4-hour fast. Serum LDL-C concentration was determined using an automated serum chemistry analyzer ADVIA^®^1800 (Siemens, Erlangen, Germany). CD81 and LDLR levels were assessed in liver by Western blot analysis, using TfR as a loading control.

#### Effect of alirocumab on LDL-C, CD81 and LDLR in Pcsk9^hum/hum^ Ldlr^+/-^ mice

A hyperlipidemic mouse model was generated by crossing homozygous humanized PCSK9 mice (*Pcsk9*^*hum/hum*^) with heterozygous *Ldlr* mice (*Ldlr* ^*+/-*^ mice) to give *Pcsk9*^*hum/hum*^ *Ldlr* ^*+/-*^ mice [27].The *Pcsk9*^*hum/hum*^ mice were generated by swapping both murine *Pcsk9* alleles with the corresponding human PCSK9 gene [27].

Blood samples were obtained from *Pcsk9*^*hum/hum*^ *Ldlr* ^*+/-*^ mice 7 days prior to antibody injection to determine LDL-C levels. On Day 0, mice were separated into groups based on LDL-C level and alirocumab or control monoclonal antibody was administered at 10 mg/kg by subcutaneous injection. Serum and livers were collected on Day 4 after antibody administration. Serum LDL-C concentration was assessed using an automated serum chemistry analyzer ADVIA^®^1800, and CD81 and LDLR levels in livers were assessed by Western blot analysis, using GAPDH as a loading control.

Levels of monoclonal antibodies in the circulation were assessed by a Human antibody Fc-region enzyme-linked immunosorbent assay.

### Data from alirocumab clinical trials

In alirocumab Phase 3 studies, antibody tests for HCV were performed at screening and at the end of the double-blind treatment period. Patients with HCV positive tests at screening were excluded from the trials. The end of the double-blind treatment period data was available for most of the safety population in the pool of placebo-controlled studies, and one half of the safety population in the pool of ezetimibe-controlled studies. A patient with a positive HCV antibody test had reflexive testing with RNA quantification to confirm HCV status.

### Statistical analysis

All values are presented as means ± standard error (SE). Statistical analyses were performed using Prism 5.0 (Macintosh Version; GraphPad Software Inc., San Diego, CA, USA). Serum parameters for *Pcsk9*^-/-^ and wild-type mice were analyzed by student’s t-test. For alirocumab treated mice LDL-C change were analyzed by two-way ANOVA with Bonferroni post-test and western blot densitometry—by student’s t-test. A threshold of p<0.05 was considered statistically significant.

## Results

### Assessment of surface and total CD81 and LDLR levels in Huh-7 cells

**Fig 1:**
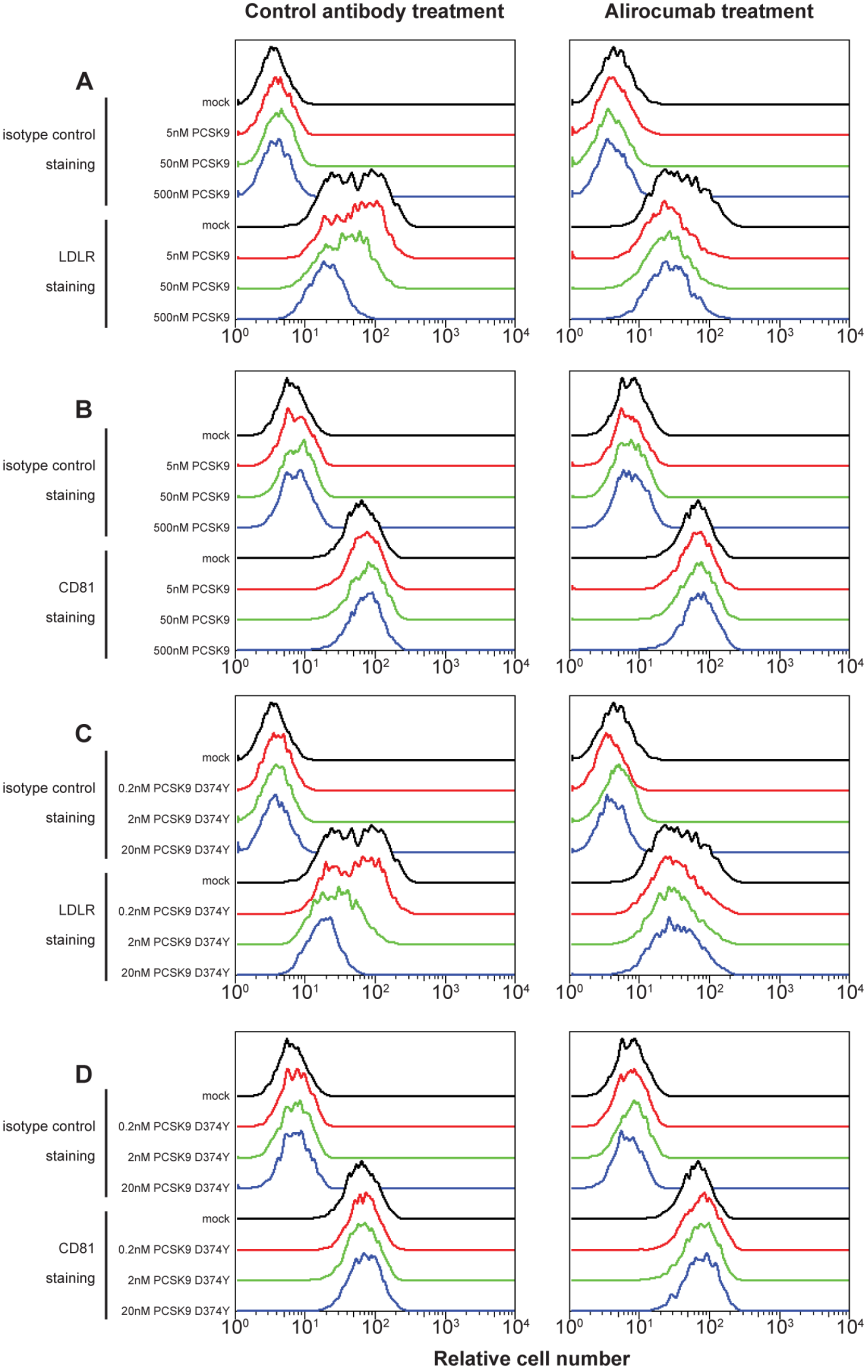
Flow cytometry quantification of surface levels of LDLR (parts A and C) and CD81 (parts B and D) on Huh-7 cells, following incubation with the indicated concentrations of wild-type PCSK9 (parts A and B) or the gain-of-function PCSK9 D374Y mutant (parts C and D) and 300 nM alirocumab (monoclonal antibody to PCSK9; right panels) or isotype control monoclonal antibody (left panels) for 6 hours. LDLR, low-density lipoprotein receptor; PCSK9, proprotein convertase subtilisin/kexin type 9.

The immortalized human hepatocarcinoma cell line used in these studies, Huh-7, endogenously expresses both LDLR and CD81, and similar to other cell culture systems, Huh-7 cells secrete low but measurable amounts of PCSK9 (data not shown). Thus, to simulate the PCSK9 levels found in human serum [28], Huh-7 cells were incubated with increasing amounts of a recombinant purified form of human PCSK9, or with the gain-of-function mutant PCSK9 D374Y, which has higher affinity for the LDLR [29]. Flow cytometry was then used to measure the amount of cell surface LDLR and CD81. As expected, increasing amounts of PCSK9 or PCSK9 D374Y resulted in a dose-dependent decrease of surface LDLR levels (Fig 1, left column). However, the surface levels of CD81 remained constant at all concentrations of PCSK9 or PCSK9 gain-of-function mutant used.

To ensure that the observed effect on LDLR was caused by PCSK9, the PCSK9-LDLR interaction was blocked using alirocumab (Fig 1, right column). As anticipated, in the presence of alirocumab, addition of soluble PCSK9 had no effect on surface LDLR levels. In addition, alirocumab had no effect on CD81, irrespective of the concentration of PCSK9 used.

**Fig 2:**
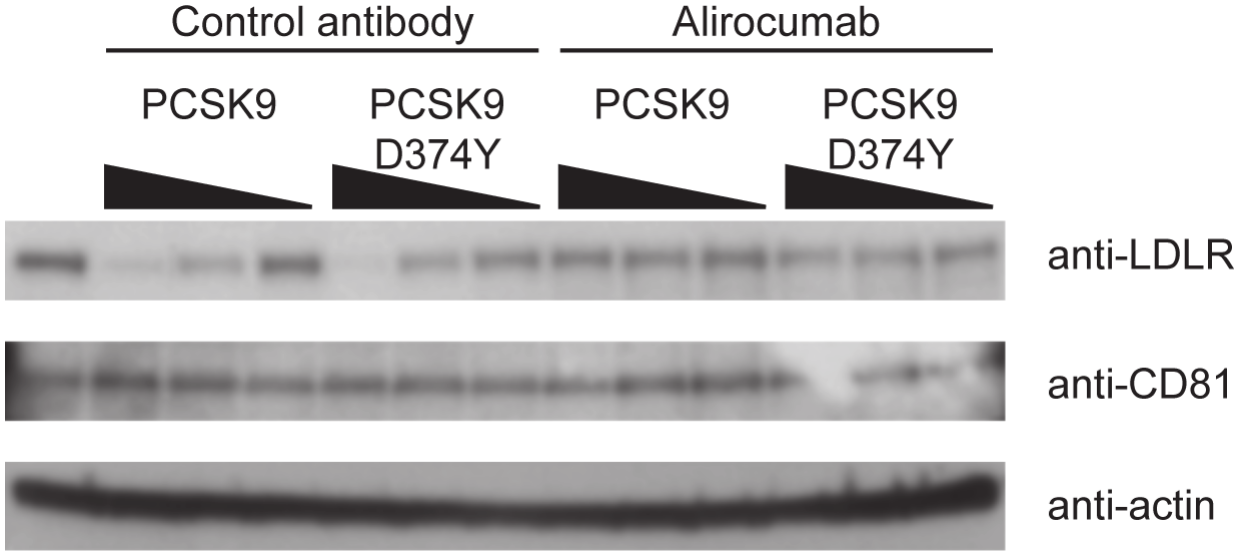
Western blot analysis on Huh-7 cells following incubation with decreasing concentrations of wild-type PCSK9 (500 nM, 50 nM and 5 nM) or gain-of-function PCSK9 D374Y mutant (20 nM, 2 nM and 0.2 nM) and 200 nM of alirocumab (monoclonal antibody to PCSK9) or isotype control monoclonal antibody for 6 hours. Lane 1 represents control cells incubated without PCSK9 protein or alirocumab/control Ab. Ab, antibody; LDLR, low-density lipoprotein receptor; PCSK9, proprotein convertase subtilisin/kexin type 9.

These results were confirmed in parallel Western blot experiments, which showed that an increase in extracellular PCSK9 concentration resulted in a decrease in total LDLR protein levels, whereas CD81 levels remained unchanged (Fig 2). Importantly, alirocumab treatment restored LDLR levels after addition of soluble PCSK9, but again had no impact on CD81 levels.

These results also confirmed that the Huh-7 cells were responsive to PCSK9 treatment: surface LDLR levels decreased as extracellular PCSK9 concentration increased, and this could be reversed in the presence of the anti-PCSK9 monoclonal antibody, alirocumab. However, unlike LDLR, CD81 levels were not affected by the presence of PCSK9 either alone or in combination with alirocumab, clearly demonstrating that extracellular PCSK9 does not have an effect on surface or total CD81 levels.

### *In vivo* relationship between PCSK9 and CD81

*In vivo* experiments were performed to further investigate the relation between PCSK9 and CD81, and the effect of antibody-mediated blockade of PCSK9 on CD81 levels.

**Fig 3:**
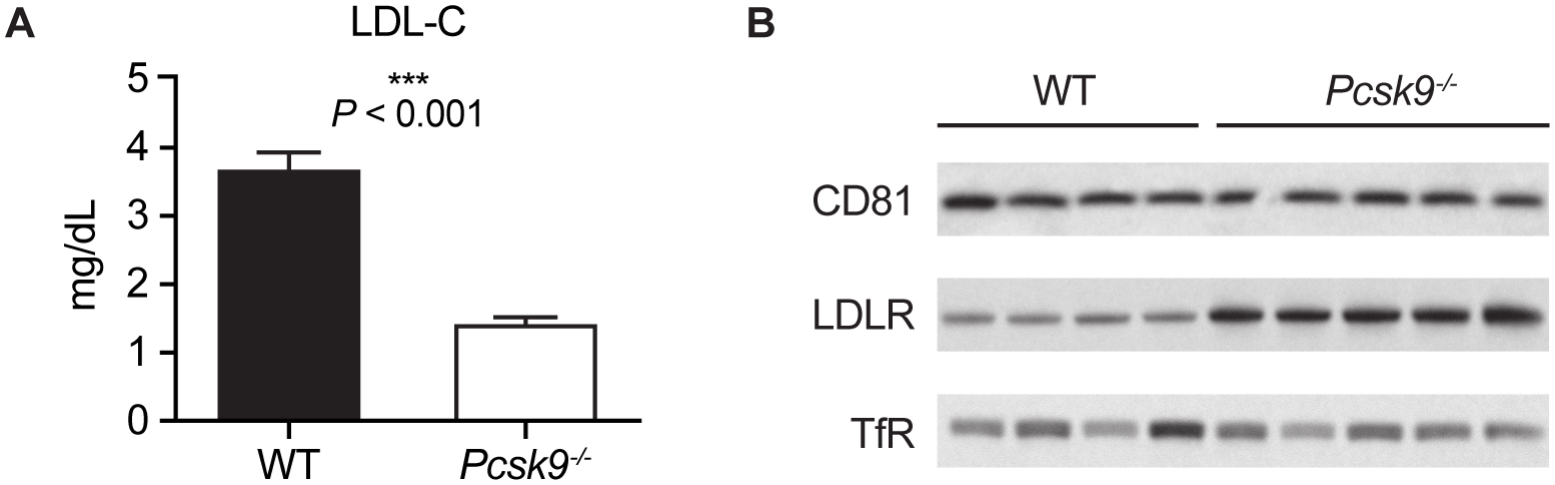
Effect of *Pcsk9* deletion on LDLR and CD81 levels in age- and sex-matched *Pcsk9*^*-/-*^ mice (n = 5) and their wild type littermates (n = 4). Shown are (A) mean ± SE serum LDL-C levels and (B) Western blot analysis of CD81 and LDLR levels in liver extracts with TfR as loading control, in animals sacrificed after a 4-hour fast. Lanes in panel B represent samples from individual mice. LDL-C, low-density lipoprotein cholesterol; LDLR, low-density lipoprotein receptor; PCSK9, proprotein convertase subtilisin/kexin type 9; SE, standard error; TfR, transferrin receptor; wt, wild type.

To examine the effect of *pcsk9* deletion on CD81 levels, *Pcsk9*^*-/-*^ mice and their wild type littermates were sacrificed, and livers and blood (serum) collected. Analysis of serum and western blot of liver extracts indicated that deletion of *pcsk9* resulted in a significant reduction in serum LDL-C concentration (Fig 3A) and an associated increase in LDLR level (~3 times), as expected, but had no effect on CD81 levels (Fig 3B).

**Fig 4:**
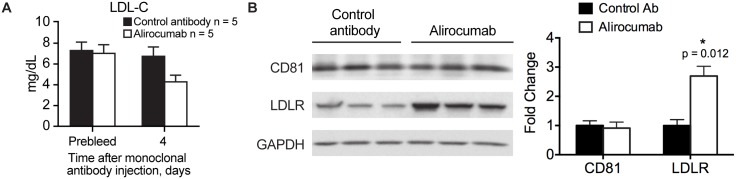
Effect of administration of alirocumab (monoclonal antibody to PCSK9) or control antibody (10 mg/kg) on LDLR and CD81 levels in hyperlipidemic *Pcsk9*^*hum/hum*^ *Ldlr* ^*+/-*^ mice. Shown are mean ± SE serum LDL-C levels (A) and Western blot analysis of CD81 and LDLR levels in liver extracts using GAPDH as loading control (B). Western blot in B was quantified using Image J. The intensities of CD81 and LDLR bands were adjusted to respective loading control for each lane and presented as a fold change from control antibody treated group. Means ± SE are shown. Serum and livers were collected on Day 4 after antibody administration. In panel B, the three columns represent three livers collected for each of the two treatments. Ab, antibody; GAPDH, glyceraldehyde 3-phosphate dehydrogenase; LDL-C, low-density lipoprotein cholesterol; LDLR, low-density lipoprotein receptor; mAb, monoclonal Ab.

The effect of alirocumab administration on LDL-C and CD81 levels was also evaluated in *Pcsk9*^*hum/hum*^ *Ldlr* ^*+/-*^ mice, with serum and livers collected four days after administration of alirocumab or an isotype-matched control antibody with irrelevant specificity. Analysis of serum samples revealed a mean 37% reduction in serum LDL-C level after administration of alirocumab compared with the isotype control monoclonal antibody (Fig 4A). The presence of monoclonal antibodies (alirocumab or isotype control) in the circulation at levels above 10 μg/ml at the time of serum/tissue collection were confirmed by a human Fc enzyme-linked immunosorbent assay (data not shown). Consistent with the findings in Pcsk9^-/-^ mice (Fig 3), administration of alirocumab led to an approximately 3-fold increase in LDLR levels but showed no effect on CD81 levels (Fig 4B).

### Effect of alirocumab on HCV cell entry

**Fig 5:**
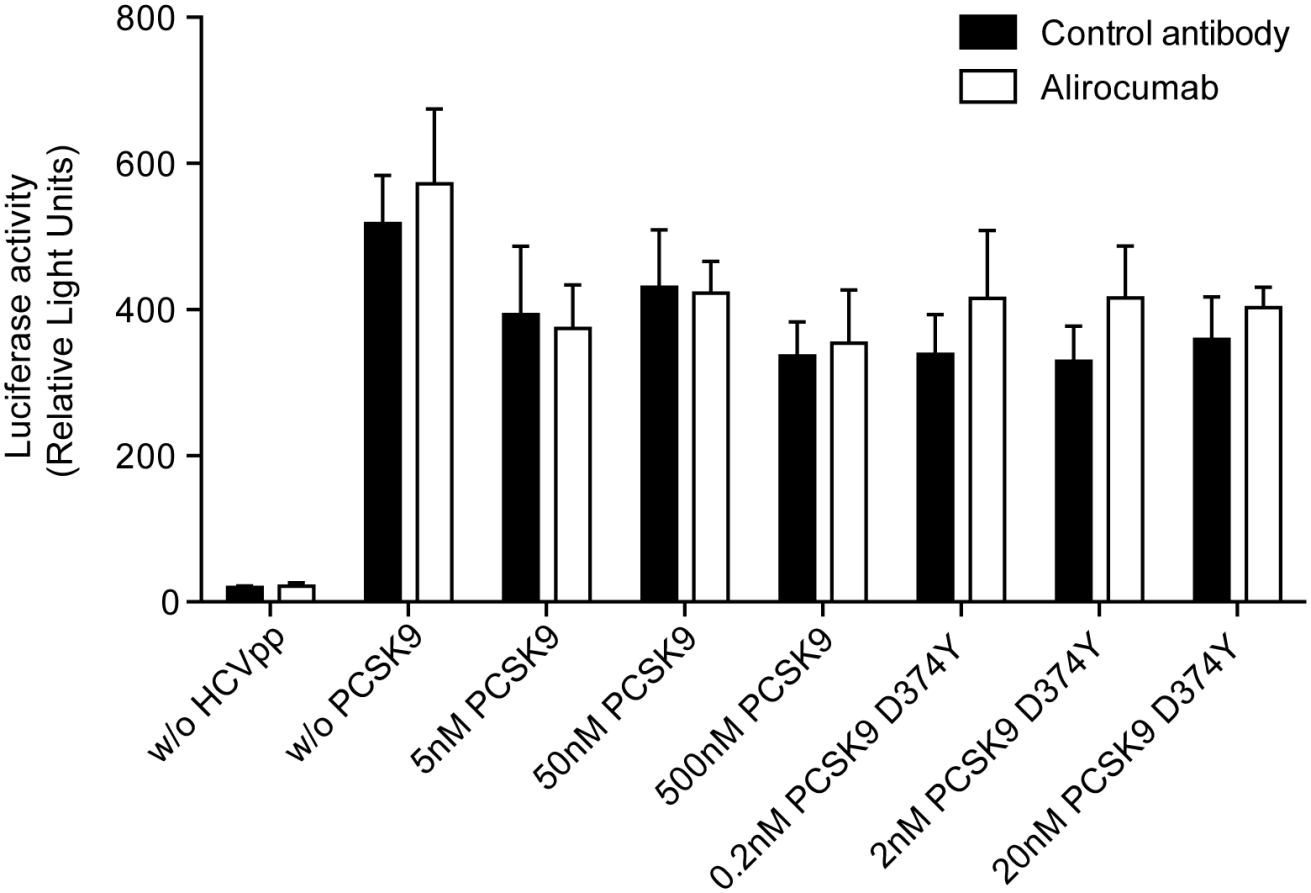
Effect of soluble PCSK9, gain-of-function PCSK9 D374Y and alirocumab on HCVpp entry. Huh-7 cells were incubated for 6 hours with the indicated concentrations of wild-type PCSK9 or the gain-of-function PCSK9 D374Y mutant and alirocumab (monoclonal antibody to PCSK9) or isotype control monoclonal antibody (n = 3 replicates per treatment). Cells were then infected with HCVpp, incubated for 48–72 hours and intracellular luciferase activity was measured. Luciferase activity is shown as relative light units. Ab, antibody; HCVpp, hepatitis C virus pseudoparticles; PCSK9, proprotein convertase subtilisin/kexin type 9; RLU, relative light units; w/o, without.

To directly assess the effect of extracellular PCSK9 and alirocumab antibody treatment on the HCV entry process, we used two well-established cell culture HCV cell entry models. The first was a system using lentiviral particles that deliver only a reporter gene to cells upon infection. When pseudotyped with the HCV glycoproteins these virions, termed HCVpp, take on the HCV cell entry pathways, and reporter expression in cells challenged with these particles is directly proportional to their ability to support this process [30, 31]. Huh-7 cells were first incubated with increasing concentrations of either wild-type or gain of function D374Y mutant recombinant PCSK9 proteins in combination with either alirocumab or its isotype control. These cells were then transduced with a luciferase-expressing HCVpp and resulting intracellular levels of luciferase were measured 48–72 hours after infection as an indirect measure of the cells’ susceptibility to HCVpp entry. We found that luciferase activity was similar regardless of whether the cells had been incubated with increasing concentrations of human wild-type PCSK9 or the D374Y mutant and/or alirocumab antibody treatment (Fig 5), indicating that the susceptibility of Huh7 cells to HCVpp is not affected by extracellular levels of PCSK9 or alirocumab.

**Fig 6:**
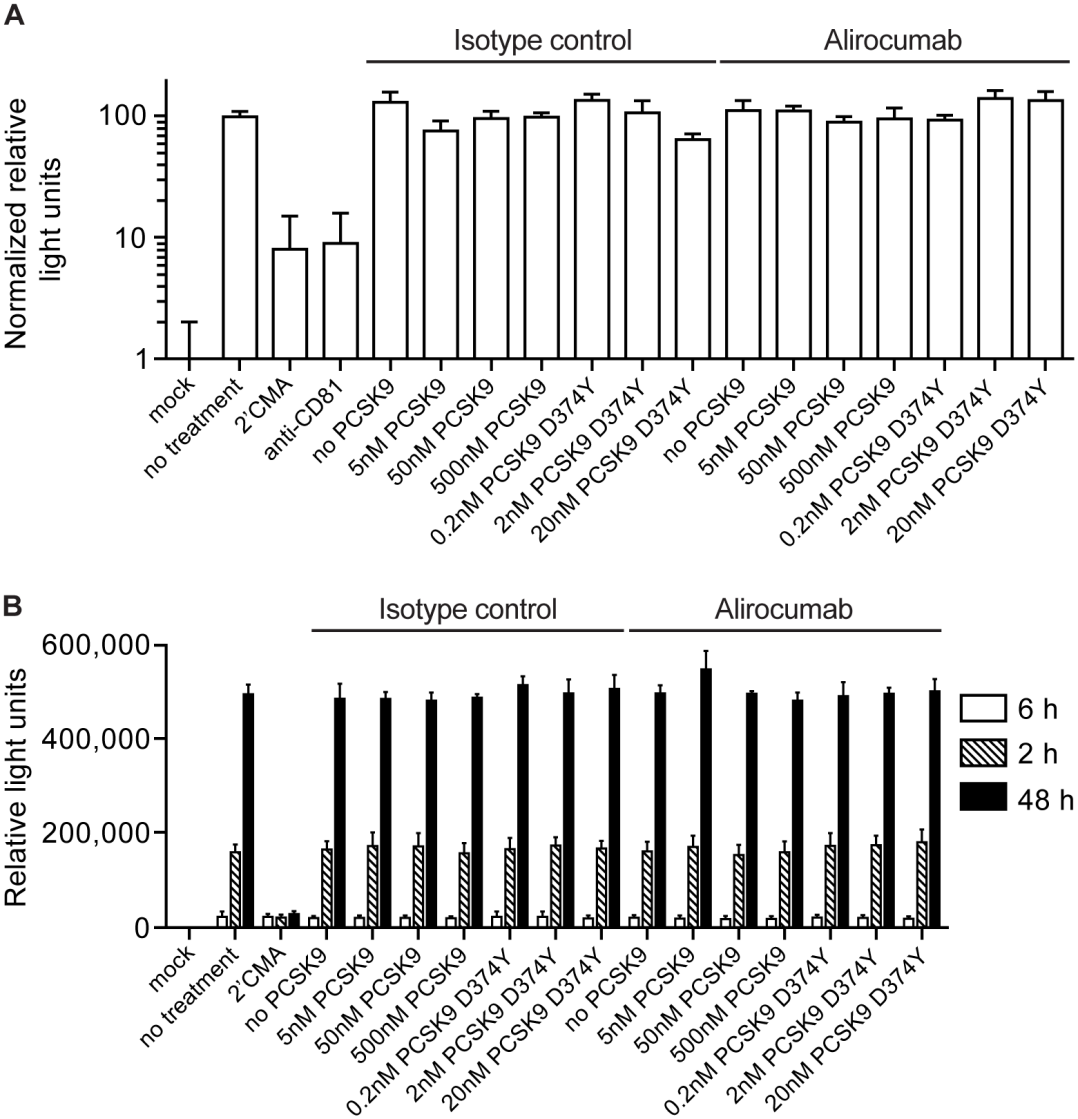
Effect of PCSK9 and alirocumab on the HCVcc full replication cycle (A) or HCV RNA replication cycle (B).

We next conducted similar infection experiments with authentic HCV infectious virus produced in cell culture. Importantly, such virus associates with apolipoproteins in a manner that HCVpp may not, and therefore may exhibit a greater dependence on LDLR for its entry into host cells. This virus, termed Jc1 [22] also encoded a secreted version of the Gaussia luciferase (GLuc) reporter protein, which could be assayed in the days following infection in the supernatant of infected cultured as a measure of HCV cell entry and intracellular replication capacity. Although the HCV polymerase inhibitor 2’CMA and CD81 specific antibodies efficiently blocked Jc1 infection, none of the protein or antibody treatments greatly affect infection with this virus. Firstly, cells treated with either alirocumab or its isotype control supported slightly greater levels of Jc1 infection, but this difference was not significantly different. Furthermore, addition of soluble PCSK9 or the hyperactive D374Y mutant, with either antibody, did not affect Jc1 infection at any concentration (Fig 6A), despite reductions in LDLR levels in target cells (Figs 1 and 2). Thus, despite a possible role for LDLR in the HCV cell entry process, infection with neither HCVpp nor Jc1 cell culture was not enhanced by alirocumab treatment.

To rule out that alirocumab influences intracellular HCV replication, we transfected cells with JFH-1 based subgenomic replicons that also express GLuc. These cells were treated with the same PCSK9 protein and antibody combinations described above, and GLuc was assayed at various times post-transfection. Although 2’CMA inhibited HCV replication and thus prevented amplification of the GLuc signal over time, all other conditions exhibited similar amplification of GLuc over two days (Fig 6B). Thus neither PCSK9 protein nor antibody affected intracellular HCV replication.

### Data on HCV infections from the alirocumab clinical development program

Data from the alirocumab Phase 3 clinical trial program were analyzed to assess rates of HCV-positive samples. This analysis included data from 10 Phase 3 trials comparing alirocumab with either placebo or ezetimibe. For purposes of the present analysis, data were analyzed in two pools according to control. Of 3182 patients treated with alirocumab, end of treatment data were available for 2281 patients; no patient was positive post-baseline for HCV ribonucleic acid (RNA) (Table 1). Of 1792 patients treated with control (placebo or ezetimibe), end of treatment data were available for 1282 patients, and again no patient was positive post-baseline for HCV RNA (Table 1). Two patients (0.1%) treated with alirocumab and two patients (0.2%) treated with controls were antibody positive for HCV during the treatment period (Table 1), indicating no evidence for an increase in susceptibility to HCV with exposure to alirocumab.

**Table 1:** Patients with post-baseline positive HCV test according to baseline status—data from alirocumab Phase 3 placebo- and ezetimibe-controlled trials.

	Placebo-controlled pool		Ezetimibe-controlled pool	
	Placebo (n = 1174)	Alirocumab (n = 2318)	Ezetimibe (n = 618)	Alirocumab (n = 864)
Baseline status—negative*/missing				
Positive RNA	0/966	0/1930	0/316	0/351
Confirmed positive ab with negative RNA	2/966 (0.2%)	2/1930 (0.1%)	0/316	0/351

*Antibody (ab) test negative or ab test positive and reflexive RNA test negative.

## Discussion

Concerns have been raised that pharmacological inhibition of PCSK9 could increase susceptibility to HCV [20] as over-expression of PCSK9 has been reported to reduce levels of CD81 [18], a major component of HCV entry into hepatocytes. However, the results of the present study indicate that although extracellular PCSK9 regulates LDLRs, it does not affect levels of CD81. This was confirmed by several methods, including administration of a native, secreted form of PCSK9 *in vitro*, or by reducing extracellular levels of PCSK9 using a monoclonal antibody, alirocumab, *in vitro* and *in vivo*.

Results of the *in vitro* experiments using human hepatocyte Huh-7 cells suggest that there is no functional relationship between the amount of the secreted, soluble form of PCSK9 and total or surface CD81 levels. Antibody blockade of PCSK9 with alirocumab also had no impact on CD81 *in vitro*. Furthermore, in a well-recognized model of HCV entry, addition of soluble PCSK9 or treatment with the alirocumab antibody was shown to have no effect on the HCV entry process.

Deletion of *Pcsk9* in mice led to increased levels of LDLR, suggesting protection from PCSK9-mediated degradation, as well as 37% reduction in circulating LDL-C levels compared with wild-type controls. Increased LDLR and reduced LDL-C were also evident in the hyperlipidemic *Pcsk9*^*hum/hum*^ *Ldlr* ^*+/-*^ mice which were administered alirocumab compared with the isotype control antibody. No changes were observed in hepatic CD81 levels in either animal model.

The immortalized human hepatocarcinoma cell line used in these studies, Huh-7, endogenously expresses both LDLR and CD81 and is typically used to study the HCV entry process [30]. However, similar to other cell culture systems, levels of PCSK9 secreted by Huh-7 cells are below the levels found in human serum [28]. Therefore, to study the effect of PCSK9 and blockade of the PCSK9/LDLR interaction under more physiological conditions, Huh-7 cells were incubated with increasing amounts of a recombinant purified form of human PCSK9, or with the gain-of-function mutant form of the protein (PCSK9 D374Y), which has higher affinity for the LDLR [29].

There were several differences in methodology between this study, and those previously reported [18, 19]. Most notably, to study the effects of increasing PCSK9 levels on Huh-7 cells we used a native, secreted form of PCSK9, which can be considered more physiologically relevant for assessing effects of monoclonal antibodies which have no impact on production of intracellular PCSK9. Differences in the observed phenotype might be attributed to the aberrant localization or increased expression used in previous studies. Alternatively, consistently with the results reported here, it is possible that unlike soluble PCSK9, the cytoplasmic and/or membrane bound form of the protein can modulate CD81 expression.

Further to the evidence from *in vitro* and *in vivo* studies reported here, data from the alirocumab clinical development program indicate no evidence for an increase in susceptibility to HCV with exposure to alirocumab. This evaluation is limited by the number of patients and duration of the studies (up to 78 weeks). The forthcoming ODYSSEY OUTCOMES study (clinicaltrial.gov identifier: NCT01663402) will provide additional and longer-term data in a larger patient population.

To conclude, these data suggest that inhibition of PCSK9 using the monoclonal antibody alirocumab has no effect on CD81, a major component of the HCV receptor complex, and does not result in increased susceptibility to HCV entry.
